# Uncommon site of Brucella endocarditis in a double discordant heart

**DOI:** 10.1093/ehjcr/ytae562

**Published:** 2024-10-26

**Authors:** Shokoufeh Hajsadeghi, Saeed Kalantari, Shayan Mirshafiee

**Affiliations:** Research Center for Prevention of Cardiovascular Disease, Institute of Endocrinology and Metabolism, Iran University of Medical Sciences, Niyayesh St, 14456-13131 Tehran, Iran; Antimicrobial Resistance Research Center, Institute of Immunology and Infectious Diseases, Iran University of Medical Sciences, Niyayesh St, 14456-13131 Tehran, Iran; Department of Cardiology, School of Medicine, Tehran University of Medical Sciences, Keshavarz blvd., 33141-14197 Tehran, Iran

## Summary

Brucella endocarditis is rare but has a high mortality rate, commonly affecting the native aortic valve, with the mitral valve occasionally involved either simultaneously or, less frequently, in isolation.^1,2^ In this case, the root of the main pulmonary artery (MPA), which is originating from the morphologic systemic left ventricle, is involved.

## Case description

A 42-year-old Afghan male was referred to Rasoul Akram Hospital Complex, Tehran, Iran with a complaint of progressive weakness and weight loss. Over the 3 months leading up to his visit, he was administered multiple intravenous and oral antibiotics empirically due to a diagnosis of fever of unknown origin. Initial laboratory examinations revealed normal white blood cell count (6400/mm³) and slightly increased C-reactive protein (10 mg/L).

Concomitantly, auscultation of high-pitch murmurs and the presence of dextrocardia in the chest X-ray prompted a cardiology consultation.

The presence of suspicious mass in MPA in transthoracic echocardiography necessitated a transoesophageal echocardiography and additional laboratory evaluations including blood culture and serologic tests.

**Figure 1:**
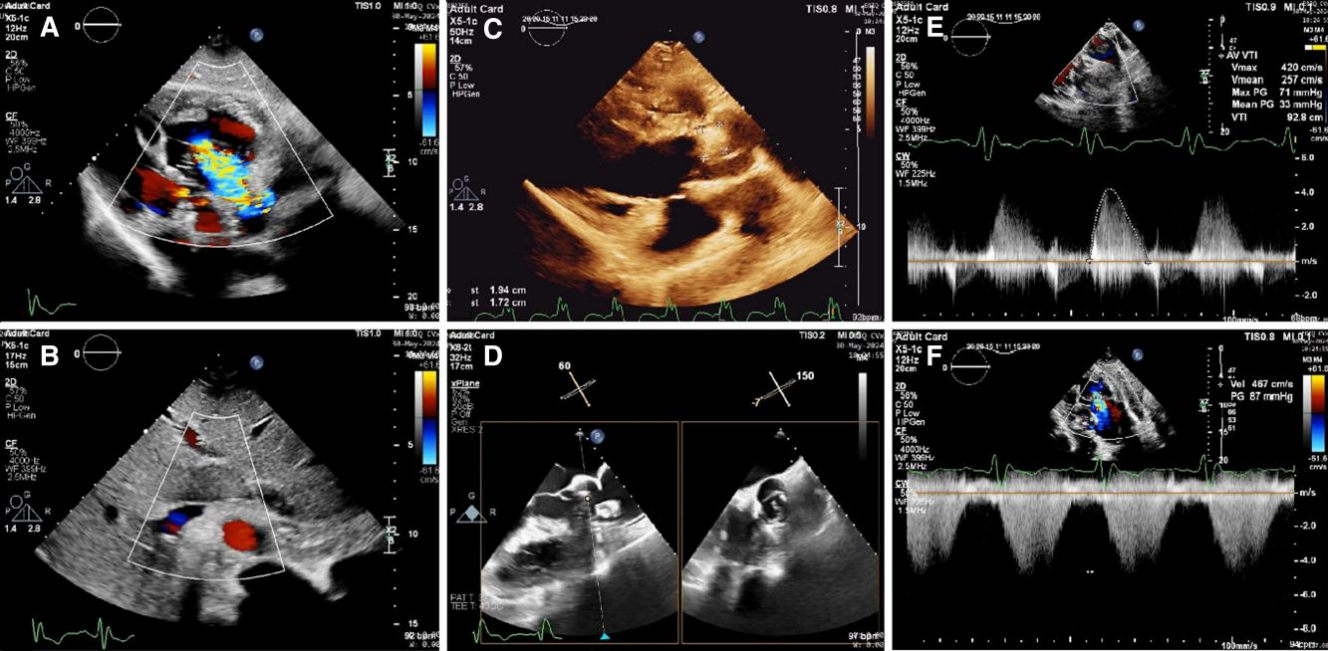
(*A*) The tricuspid valve is observed in a more apically positioned view, demonstrating severe regurgitation. The apex's orientation towards the right side is indicative of dextrocardia. (*B*) A left-sided aorta and a right-sided inferior vena cava indicate situs solitus. (*C*) A mobile mass measuring 1.92 × 1.74 cm is observed attached to the wall of the main pulmonary artery. (*D*) Biplane view demonstrating mobile mass protruding into the pulmonary valve. (*E*) Peak pressure gradient of 71 mmHg via pulmonary valve indicative of severe pulmonary valve stenosis. (*F*) Tricuspid regurgitation gradient of 87 mmHg.

The diagnosis of congenitally corrected transposition of the great arteries with pulmonary valve stenosis was established through the identification of the transposition of the aorta, positioned anteriorly and to the left of the pulmonary artery, in conjunction with the transposition of the morphological left and right ventricles, and a pulmonary valve peak pressure gradient of 71 mmHg (Supplementary material online, *Video S1*). Noteworthy was the identification of a 1.92 ∗ 1.74 cm mobile mass on the atrial side of the pulmonary valve, which was connected to the root of the MPA (*Figure 1*).

The 48-h blood cultures returned negative results; however, serological evaluation suggested a diagnosis of brucellosis, supported by the patient's history of consuming raw dairy products (Wright ≥ 1/640, Coombs Wright ≥ 1/640, and 2-mercaptoethanol Brucella agglutination (2ME) titre ≥ 1/320 by standard tube agglutination test). Due to the endemicity of Brucella in our geographical area and the presence of strongly positive serological tests, and in accordance with the presence of two major Duke’s criteria, diagnosis of Brucella endocarditis was established for the patient.^3^

Given the complex anatomy, high-risk cardiac surgery, and the patient’s preference, we opted for at least 3 months of antibiotic treatment with doxycycline 100 mg bid, rifampin 600 mg daily, and ciprofloxacin 500 mg bid.

## Supplementary Material

ytae562_Supplementary_Data

## Data Availability

The data can be requested from the corresponding author, as they are not publicly accessible due to privacy or ethical restrictions.

## Supplementary material

**Consent:** The authors confirm that written consent for the submission and publication of this work has been duly obtained from the patient, in accordance with the COPE guidelines.

**Funding:** None declared.
